# Are Long Noncoding RNAs New Potential Biomarkers in Gastrointestinal Stromal Tumors (GISTs)? The Role of H19 and MALAT1

**DOI:** 10.1155/2019/5458717

**Published:** 2019-11-15

**Authors:** Giuseppe Badalamenti, Nadia Barraco, Lorena Incorvaia, Antonio Galvano, Daniele Fanale, Daniela Cabibi, Valentina Calò, Giuseppe Currò, Viviana Bazan, Antonio Russo

**Affiliations:** ^1^Department of Surgical, Oncological, and Oral Sciences, Section of Medical Oncology, University of Palermo, Palermo, Italy; ^2^Department of Sciences for the Promotion of Health and Mother and Child Care, Anatomic Pathology, University of Palermo, Palermo, Italy; ^3^Department of Surgical, Oncological and Oral Sciences, Section of Oral Medicine, University of Palermo, Palermo, Italy; ^4^Department of Biomedicine, Neuroscience and Advanced Diagnostics, University of Palermo, Palermo, Italy

## Abstract

Long noncoding RNAs (lncRNAs) are emerging as key regulators of genetic and epigenetic networks, and their deregulation may underlie complex diseases, such as carcinogenesis. Several studies described lncRNA alterations in patients with solid tumors. In particular, HOTAIR upregulation has been associated with tumor aggressiveness, metastasis, and poor survival in gastrointestinal stromal tumor (GIST) patients. We analyzed expression levels of other lncRNAs, H19 and MALAT1, in FFPE tissue specimens from 40 surgically resected and metastatic GIST patients, using real-time PCR analysis. H19 and MALAT1 were both upregulated in 50% of GIST patients. MALAT1 lncRNA expression levels seem to be correlated with *c-KIT* mutation status. The percentage of both H19 and MALAT1 upregulation was significantly higher in patients with time to progression (TTP) < 6 months as compared to patients with TTP > 6 months. The median TTP was significantly lower in patients with both H19 and MALAT1 lncRNA upregulation as compared to those with lncRNA downregulation. These data suggest a potential role for both H19 and MALAT1 lncRNAs as prognostic biomarker for the clinical selection of the best candidate to first-line treatment with imatinib.

## 1. Introduction

Gastrointestinal stromal tumors (GISTs) are considered as a paradigm of molecular biology in solid tumors. Mutational analysis for receptor tyrosine kinase (*c-KIT*) and platelet-derived growth factor receptor alpha (*PDGFRα*) genes has a predictive value for sensitivity to targeted therapy. For GIST patients with localized disease who underwent surgical treatment, the evaluation of *c-KIT*/*PDGFRα* molecular alterations together with other clinicopathological factors, including tumor site/size, mitotic rate, and proliferation index, is crucial to predict the potential risk of recurrence and ultimately decide if patients are candidate to receive adjuvant therapy with imatinib mesylate [1]. Tumor rupture is an additional adverse prognostic factor. As regards patients with advanced disease harboring *c-KIT* activating mutations, imatinib represents the standard first-line treatment, while sunitinib and regorafenib are two multitarget tyrosine kinase inhibitors (TKIs) usually administered after the failure of imatinib therapy. In recent years, epigenetic studies are emerging as a tool to achieve relevant information about GIST biology and investigate new potential biomarkers with diagnostic, prognostic, and predictive value [2].

Several data showed that new classes of noncoding RNAs (ncRNAs), including long noncoding RNAs (lncRNA), are essential components of gene regulatory networks [3–5]. LncRNAs belong to a class of regulatory RNA noncoding for proteins that represent approximately 1.5% of the eukaryotic genome, almost entirely transcribed [6–8]. The NONCODE human lncRNA database annotated 527,336 transcripts that are antisense, intergenic, sense intronic, and processed transcript [9]. Different from other transcriptome and epigenome datasets, the lncRNAs are generated through a molecular pathway similar to that used for protein-coding genes [10]. LncRNAs have been arbitrarily defined, according to their size, as transcribed RNA molecules greater than 200 nucleotides (nt) in length in their mature form. In contrast to the small ncRNAs (siRNAs, miRNAs, and piRNAs), which are highly conserved in commonly studied species and act as negative regulator of gene expression [11, 12], lncRNAs are modestly conserved and regulate gene expression through mechanisms that are mostly poorly understood [13–18].

In the current scenario, characterized by a spasmodic research of new biomarkers and the advent of advanced technologies, the lncRNAs represent a new, valid, and largely unexplored field of investigation. Thanks to their structure, they play a critical role in a plethora of biological functions at transcriptional, posttranscriptional, and translation level, including also epigenetic processes [19, 20].

Since lncRNAs regulate several biological processes, their overexpression may be essential in the switch towards pathological conditions [21, 22]. They may be involved in the development of different human diseases, such as cancer, functioning as pro-oncogenic and/or tumor suppressor factors, by modulating tumor initiation, progression, and metastatic pathways [23–26]. Among the different lncRNAs identified in solid tumors, H19 and MALAT1 are among those better studied and characterized [27–29]. LncRNA H19 has been one of the first to be identified and is mostly expressed in the embryonic and fetal tissues [30]. The loss of H19 maternally expressed gene and its expression alteration were observed in different solid tumors [31]. Its dysregulation has been correlated with worse survival, poor disease-free survival (DFS), histological grade, positive lymph node metastasis, and advanced TNM stage [27]. Also, in plasma of preoperative patients with gastric cancer (GC), the H19 plasma levels were higher than that of healthy controls. Conversely, there were no differences in H19 expression between cancerous tissues and paired noncancerous tissues [32]. Results from a meta-analysis showed that high H19 expression levels were inversely correlated with OS and prognosis in many types of cancer, suggesting a potential negative prognostic role for this biomarker [27].

MALAT1 is the most expressed among lncRNAs and acts as an oncogenic factor. In stress condition, it has been found to be upregulated in common sites of metastasis from different solid tumors, mainly in lung cancer [33]. In lung cancer, it plays a role as a negative prognostic marker [34, 35]. As shown by a recent meta-analysis, high expression of MALAT1 positively correlated with worse patient prognosis also in breast, ovarian, colon, pancreatic, and digestive cancers [36]. Moreover, MALAT1 plasma levels were similar to those of healthy controls in GC patients [32].

Few studies investigated the *in vivo* and *in vitro* expression of lncRNAs in GISTs. In this scenario, the identification of lncRNA as new potential diagnostic, prognostic, and predictive molecular biomarkers represents a new challenge for current translational research in GISTs [37]. We analyzed the *in vivo* expression levels of lncRNAs H19 and MALAT1 in tissue specimens from both surgically resected and metastatic GIST patients. We subsequently investigated their potential prognostic role in relation to the clinicopathological features and response to TKI treatment.

## 2. Results

### 2.1. Clinical Characteristics of the Patients

Forty patients with histopathological diagnosis of GIST were included in the study. Twenty-five patients (62.5%) had localized disease at diagnosis and 15 (37.5%) advanced disease. *C-KIT* and *PDGFRα* mutations were detected in 27 out of 40 (67.5%) patients, 17 patients of which were with localized disease and 10 patients with metastatic disease. 8 out of 25 patients (32%) with localized disease harboring *c-KIT* mutations had a high risk of relapse and, therefore, have received adjuvant treatment with imatinib for 3 years. Fifteen patients with metastatic disease received first-line therapy with imatinib until progression disease (PD) or unacceptable toxicity (Table 1). Other clinical and pathological characteristics of patients included in this study are summarized in Supplementary [Supplementary-material supplementary-material-1].

### 2.2. Expression of lncRNAs H19 and MALAT1 in GISTs

**Figure 1:**
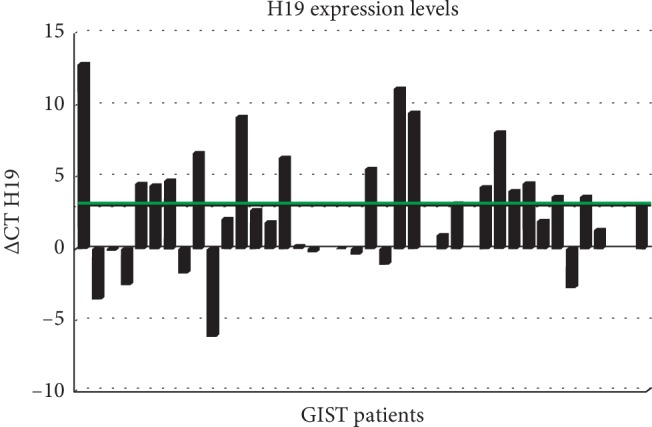
H19 expression levels in paraffin tissue of GIST samples. Expression levels are represented as delta CT. The median value of delta CT is illustrated as a green line.

Among the 40 patients, 34 were evaluable for lncRNA H19 expression analysis in tumor tissue. The median ΔCT for H19 expression was 3 (range: –6–+13) in the overall GIST population. Considering as cutoff value the median ΔCT value of 3, 17 patients (50%) showed median ΔCT higher than 3 (Figure 1). The association between lncRNA H19 expression levels and patients' clinicopathological characteristics, including tumor size, mitotic index, stage at diagnosis, class of risk according to Miettinen's criteria, and *c-KIT* mutation status are described in Table 2.

**Figure 2:**
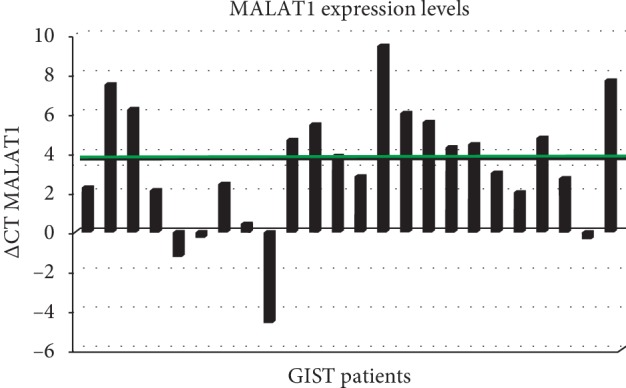
MALAT1 expression levels in paraffin tissue of GIST samples. Expression levels are represented as delta CT. The median value of delta CT is illustrated as a green line.

Among the 40 patients, 24 were evaluable for lncRNA MALAT1 expression analysis in tumor tissue. The median ΔCT for MALAT1 expression was 3.5 (range: –5–+9) in the overall GIST population. Considering as cutoff value the median ΔCT value of 3.5, half of the patients showed median ΔCT lower than 3.5 (Figure 2). The association between lncRNA MALAT1 expression levels and patients' clinicopathological characteristics, including tumor size, mitotic index, stage at diagnosis, class of risk according to Miettinen's criteria, and *c-KIT* mutation status are described in Table 3. High expression levels of MALAT1 in paraffin tumor tissue have been detected in 8 out of 16 (50%) GIST patients harboring *c-KIT* mutations as compared to 1 out of 4 (25%) wild-type patients.

### 2.3. LncRNA Expression and Treatment Efficacy

Among 40 patients included in our study, only 15 patients with advanced disease who received imatinib as first-line treatment were considered for efficacy analysis. In order to obtain homogeneous groups, we selected 5 patients with time to progression (TTP) > 6 months and 5 patients with TTP < 6 months. High expression levels of H19 have been found in 3 out of 5 (60%) patients with TTP < 6 months as compared to 2 out of 5 (40%) patients with TTP > 6 months. High expression levels of MALAT1 have been found in 3 out of 5 (60%) patients with TTP < 6 months compared to 2 out of 5 (40%) patients with TTP > 6 months.

The median TTP was significantly lower in patients with “higher” lncRNA H19 as compared to those with “lower” lncRNA H19 (8.4 vs. 20.2 months; *p* value: 0.048). Similarly, the median TTP was significantly lower in patients with “higher” lncRNA MALAT1 as compared to those with “lower” lncRNA MALAT1 (6 vs. 13 months; *p* value: 0.042).

## 3. Discussion

Several evidences showed lncRNA dysregulation in preclinical tumor models, suggesting their potential involvement in cancer development. We assessed the expression of H19, HOTAIR, and MALAT1 as they were better studied among the different lncRNAs identified in solid tumors as a hallmark of poor prognosis. They may contribute to the carcinogenesis as oncogenic and/or tumor suppressor factors, by playing essential biological functions, including chromatin modification, and transcriptional and posttranscriptional processes [38, 39]. Dysregulation of H19, HOTAIR, and MALAT1 was observed in many types of cancer [40, 41]. Their high expression level was associated with tumor cell proliferation, invasion, and metastasis, suggesting that these lncRNAs may be potential prognostic biomarkers. Indeed, studies *in vitro* showed that their knockdown could inhibit invasions and metastasis [36, 42, 43]. Therefore, the identification of lncRNAs as new biomarkers for clinical use could represent an important finding in the context of rare tumors as GISTs. Although lncRNAs have already attracted the attention of the scientific community, however, the clinical significance of the lncRNA expression has been not yet understood. In this study, for the first time, we found that both lncRNAs H19 and MALAT1 showed high expression level in tumor specimens of GIST patients. Particularly, the higher expression level of MALAT1 seems to be associated with the *c-KIT* mutation status. Indeed, the percentage of “higher” MALAT1 was found in *c-KIT* mutated *versus* wild-type GIST. In addition to the known negative prognostic role of *c-KIT* mutations in GIST, we evaluated also a potential prognostic role of both H19 and MALAT1 lncRNA in our population. As shown by the analysis of TTP in the subgroup of patients with advanced disease who received first-line therapy with imatinib, the percentage of both H19 and MALAT1 “higher” was significantly incremented in patients with TTP <6 months as compared to patients with TTP >6 months. Although the low number of patients included, these data would suggest a potential negative prognostic role of both H19 and MALAT1 lncRNAs “higher,” which seems to be associated with an “early” PD to imatinib. Also, we demonstrated a significantly lower median TTP in patients with H19 as well as MALAT1 “higher” as compared to those with “lower” lncRNAs. According to these evidences, the evaluation of lncRNA expression could allow to select, among all *c-KIT*-mutated GIST patients eligible to receive first-line therapy with imatinib, those who could really benefit from this treatment reserving another more effective therapy to the others, with interesting implications for their clinical management. However, the low number of patients included in the study and the heterogeneity of their clinicopathological characteristics limits the statistical significance of our analysis as well as the scientific reliability of the results, which need to be explored and confirmed in prospective studies including larger patients' cohort.

In conclusion, the results of this study first showed high expression level of both H19 and MALAT1 lncRNAs in FFPE GIST specimens. The expression levels of MALAT1 lncRNA seem to be correlated with *c-KIT* mutational status. The expression levels of both H19 and MALAT1 lncRNAs seem to be associated with patients' prognosis and clinical response to imatinib in advanced GIST harboring *c-KIT* mutations, suggesting a potential role for the clinical selection of the best candidate to first-line treatment. Recent evidences identified lncRNAs in plasma exosome or also in complex with circulating microRNAs. Although these studies clearly demonstrated that there are many functional circulating lncRNA, key questions remain to be solved. As recently shown in other tumors, it would be also interesting to evaluate the expression levels of circulating lncRNAs in plasma of GIST patients and compare them with the results obtained in tumor tissue. In the last years, groups of researchers have walked several roads to identify biomarkers which could help the early detection and screening, choice of surgical or medical treatments, and monitoring during the follow-up period. This represents an interesting contribution to research which aims to further personalize the management and treatment of GIST patients.

## 4. Materials and Methods

### 4.1. Population Study

A total of 40 patients with diagnosis of GIST were enrolled in this institutional translational research study: 25 patients with localized disease and 15 patients with advanced disease. All methods were carried out in accordance with relevant guidelines and regulations, and all experimental protocols were approved by the ethical committee of the university-affiliated Hospital Policlinico “Paolo Giaccone” of Palermo (Italy). Written informed consent was obtained from all participant subjects before recruitment in the study and specimen collection. All patients with diagnosis of metastatic disease harboring *c-KIT* activating mutations received oral imatinib mesylate at 400 mg daily until progression disease or unacceptable toxicity. Among the patients with localized disease subjected to surgical treatment, only those defined at high risk of recurrence according to the risk definition system proposed by Miettinen's et al. received imatinib mesylate at 400 mg daily for 3 years. All the patients underwent a CT scan every 6 months, and responses were classified according to RECIST and Choi criteria.

### 4.2. Tumor Samples

The disease formalin-fixed paraffin-embedded (FFPE) tissue from GIST patients was collected. The tumor samples were analyzed for *c-KIT* exon 9, 11, 13, and 17 and PDGRF*α* exon 12, 14, and 18 mutations. Further tissue sections were used for gene expression analysis.

### 4.3. DNA Preparation and Mutation Screening

Genomic DNA was extracted from FFPE tissues, using the QIAamp DNA FFPE Tissue Kit (Qiagen). To detect hotspot mutations, we amplified exons 9, 11, 13, 17, and 18 of the *c-KIT* gene by PCR in a preparation of genomic DNA. The primer sequences are listed in Table 4. We purified PCR products with PureLink® PCR Purification Kit (Thermo Fisher Scientific) and directly sequenced them using BigDye XTerminator® Purification Kit (Thermo Fisher Scientific) through the ABI 3130 XL Genetic Analyzer automated sequencer (Applied Biosystems). Sequence data were analyzed using Sequencing Analysis software 5.2 (Applied Biosystems).

### 4.4. LncRNA Isolation

FFPE tissue samples were deparaffinized and underwent total RNA and lncRNA extraction using miRNeasy FFPE Kit (Qiagen Inc., Valencia, CA, USA) according to the manufacturer's instructions. LncRNA yield was determined through a Qubit™ 3.0 Fluorometer (Thermo Fisher Scientific), and the quality was assessed by agarose gel electrophoresis. The lncRNA concentration and quality were assessed with the Bioanalyzer 2100 (Agilent Technologies, CA) using the Agilent Small RNA Analysis kit (Agilent, CA).

### 4.5. Quantitative Real-Time PCR (qRT-PCR) Analysis

Quantitative real-time PCR was used to measure lncRNA expression levels in GIST samples. 500 nanograms of total RNA was reverse-transcribed using High-Capacity cDNA Reverse Transcription Kit (Thermo Fisher Scientific) according to the manufacturer's instructions. The following Applied Biosystems assays were used for TaqMan analysis: ID Hs00399294_g1 for *H19*; ID Hs00273907_s1 for MALAT1. The qRT-PCR was performed through the Applied Biosystems 7900HT fast RT-PCR system, and data were collected and analyzed using ABI SDS version 2.3. Triplicate reactions were performed on all samples. To normalize qRT-PCRs, parallel reactions were run on each sample for *GAPDH* control gene (assay ID Hs03929097_g1). The lncRNA expression levels were determined using a comparative CT method and were reported as “delta CT.”

### 4.6. Statistical Analysis

Statistical analysis was performed using Microsoft Excel and GraphPad Prism software (GraphPad software, CA). The median delta CT was calculated in GIST patients and was used to define lncRNA upregulation in analyzed tumor tissues. Patients were classified as “upregulated” if they had delta CT higher than the median value observed in the overall population, or “downregulated” if they had delta CT lower than the median value observed in the overall population. To compare two independent samples, the chi-square test was used for intergroup comparison of categorical variables, while the Mann–Whitney test was used for statistical analysis of continuous variables. TTP was calculated from the date of admission to the date of first radiologic progression. Survival analysis was performed using the Kaplan–Meier method, providing median and *p* value. A *p* value < 0.05 was used as a threshold for statistical significance.

## 5. Conclusions

In our work, we suggest a potential, opposite, prognostic value of both H19 and MALAT1 lncRNAs in GIST patients. Further analyses are needed to confirm these data and evaluate the potential role of such lncRNAs as prognostic and/or predictive biomarker.

## Figures and Tables

**Table 1:** Type of mutations, stages of disease (localized or metastatic), and setting of imatinib treatment (adjuvant or first line) of GIST patients.

*Mutation analysis*		
Wild-type		13
Mutated	*c-KIT*	25
	PDGFR*α*	2

*Onset*		
Localized		25
Metastatic		15

*Imatinib, 400 mg*		
Mutated *c-KIT*	Adjuvant	8
	First line	10

**Table 2:** Association between clinicopathological features and lncRNA H19 expression levels.

Variable	H19 expression level	
	Higher	Lower
Patients		
34	17	17

Mitotic rate: *n* (%)		
<5/50 HPF: 9	6	3
≥5/50 HPF: 11	4	7

Tumor size: *n* (%)		
<5 cm: 12	6	6
≥5 cm: 8	4	4

Tumor site		
Stomach/small intestine: 22	11	11
Colon-rectum: 2	2	0

Risk classification (Miettinen's criteria)		
Low risk/very low risk: 7	5	2
Intermediate/high risk: 7	4	3

Onset		
Localized: 24	11	13
Metastatic: 10	6	4

Mutation analysis		
Wild-type: 7	4	3
Mutated: 21	10	11

**Table 3:** Association between clinicopathological features and lncRNA MALAT1 expression levels.

Variable	MALAT1 expression level	
	Higher	Lower
Patients		
24	12	12

Mitotic rate		
<5/50 HPF: 0		
≥5/50 HPF: 6	6	1

Tumor size		
<5 cm: 1	1	
≥5 cm: 6	5	1

Tumor site		
Stomach/small intestine: 9	5	4
Colon-rectum: 2		2

Risk classification (Miettinen's criteria)		
Low risk/very low risk: 4	2	2
Intermediate/high risk: 4	3	1

Onset		
Localized: 14	6	8
Metastatic:10	5	5

Mutational analysis		
Wild-type: 4	1	3
Mutated: 16		8

**Table 4:** Primers used for the analysis of *c-KIT* and *PDGFRα* genetic aberrations.

Exon	Primer set
9	F:5′-AGC CAG GGC TTT TGT TTT CT-3′
	R:5′-CAG AGC CTA AAC ATC CCC TTA-3′

11	F:5′-CCT TTG CTG ATT GGT TTC GT-3′
	R:5′-ACC CAA AAA GGT GAC ATG GA-3′

13	F:5′-GTT CCT GTA TGG TAC TGC ATG CG-3′
	R:5′-CAG TTT ATA ATC TAG CAT TGC C-3′

17	F:5′-CTG AAT ACT TTA AAA CAA AAG TAT TGG-3′
	R:5′-TTA TGA AAA TCA CAG GAA ACA ATT T-3′

12	F:5′-AAG CTC TGG TGC ACT GGG ACT T-3′
	R:5′-ATT GTA AAG TTG TGT GCA AGG GA-3′

14	F:5′-CAG GAT TAG TCA TAT TCT TGG TTT TT-3′
	R:5′-TTC TAT TCC CTG CCA TGT GT-3′

18	F:5′-TAC AGA TGG CTT GAT CCT GAG T-3′
	R:5′-AGT GTG GGA GGA TGA GCC TG-3′

## Data Availability

The data used to support the findings of this study are available from the corresponding author upon request.

## Supplementary Materials

Supplementary Materials Supplementary Table 1: clinical features of the GIST samples. Majority of the patients were males (26; 65%). The median age of the patients was 59 years. Majority of the patients had a mitotic rate <5/50 HPF, tumor size <5 cm, and gastric and intestinal site.

## References

[1] Badalamenti G., Rodolico V., Fulfaro F. (2007). Gastrointestinal stromal tumors (GISTs): focus on histopathological diagnosis and biomolecular features. *Annals of Oncology*.

[2] Xu W., Yang Z., Lu N. (2016). From pathogenesis to clinical application: insights into exosomes as transfer vectors in cancer. *Journal of Experimental & Clinical Cancer Research*.

[3] Mirabella A. C., Foster B. M., Bartke T. (2015). Chromatin deregulation in disease. *Chromosoma*.

[4] Fanale D., Barraco N., Listì A., Bazan V., Russo A. (2016). Non-coding RNAs functioning in colorectal cancer stem cells. *Advances in Experimental Medicine and Biology*.

[5] Fanale D., Castiglia M., Bazan V., Russo A. (2016). Involvement of non-coding RNAs in chemo- and radioresistance of colorectal cancer. *Advances in Experimental Medicine and Biology*.

[6] Carninci P., Kasukawa T., Katayama S. (2005). The transcriptional landscape of the mammalian genome. *Science*.

[7] Pennisi E. (2010). Shining a light on the genome’s “dark matter”. *Science*.

[8] Wang K. C., Chang H. Y. (2011). Molecular mechanisms of long noncoding RNAs. *Molecular Cell*.

[9] Zhao Y., Li H., Fang S. (2016). NONCODE 2016: an informative and valuable data source of long non-coding RNAs. *Nucleic Acids Research*.

[10] Derrien T., Johnson R., Bussotti G. (2012). The GENCODE v7 catalog of human long noncoding RNAs: analysis of their gene structure, evolution, and expression. *Genome Research*.

[11] Fanale D., Amodeo V., Bazan V. (2016). Can the microRNA expression profile help to identify novel targets for zoledronic acid in breast cancer?. *Oncotarget*.

[12] Cabibi D., Caruso S., Bazan V. (2016). Analysis of tissue and circulating microRNA expression during metaplastic transformation of the esophagus. *Oncotarget*.

[13] Bernstein E. (2005). RNA meets chromatin. *Genes & Development*.

[14] Bracken A. P., Helin K. (2009). Polycomb group proteins: navigators of lineage pathways led astray in cancer. *Nature Reviews Cancer*.

[15] Faghihi M. A., Wahlestedt C. (2009). Regulatory roles of natural antisense transcripts. *Nature Reviews Molecular Cell Biology*.

[16] Mercer T. R., Dinger M. E., Mattick J. S. (2009). Long non-coding RNAs: insights into functions. *Nature Reviews Genetics*.

[17] Wilusz J. E., Sunwoo H., Spector D. L. (2009). Long noncoding RNAs: functional surprises from the RNA world. *Genes & Development*.

[18] Paladini L., Fabris L., Bottai G., Raschioni C., Calin G. A., Santarpia L. (2016). Targeting microRNAs as key modulators of tumor immune response. *Journal of Experimental & Clinical Cancer Research*.

[19] Li Y., Chen J., Zhang J. (2015). Construction and analysis of lncRNA-lncRNA synergistic networks to reveal clinically relevant lncRNAs in cancer. *Oncotarget*.

[20] Rinn J. L., Chang H. Y. (2012). Genome regulation by long noncoding RNAs. *Annual Review of Biochemistry*.

[21] Yoon J.-H., Abdelmohsen K., Gorospe M. (2014). Functional interactions among microRNAs and long noncoding RNAs. *Seminars in Cell & Developmental Biology*.

[22] Bartonicek N., Maag J. L. V., Dinger M. E. (2016). Long noncoding RNAs in cancer: mechanisms of action and technological advancements. *Molecular Cancer*.

[23] Prensner J. R., Chinnaiyan A. M. (2011). The emergence of lncRNAs in cancer biology. *Cancer Discovery*.

[24] Taft R. J., Pang K. C., Mercer T. R., Dinger M., Mattick J. S. (2010). Non-coding RNAs: regulators of disease. *The Journal of Pathology*.

[25] Yu G., Lin J., Liu C., Hou K., Liang M., Shi B. (2017). Long non-coding RNA SPRY4-IT1 promotes development of hepatic cellular carcinoma by interacting with ERR*α* and predicts poor prognosis. *Scientific Reports*.

[26] Peng Z., Wang J., Shan B. (2017). Genome-wide analyses of long noncoding RNA expression profiles in lung adenocarcinoma. *Scientific Reports*.

[27] Liu F., Pan H., Xia G., Qiu C., Zhu Z. (2016). Prognostic and clinicopathological significance of long noncoding RNA H19 over expression in human solid tumors: evidence from a meta-analysis. *Oncotarget*.

[28] Gupta R. A., Shah N., Wang K. C. (2010). Long non-coding RNA HOTAIR reprograms chromatin state to promote cancer metastasis. *Nature*.

[29] Li Z. (2011). MALAT-1: a long non-coding RNA and its important 3′ end functional motif in colorectal cancer metastasis. *International Journal of Oncology*.

[30] Tabano S., Colapietro P., Cetin I. (2014). Epigenetic modulation of theIGF2/H19 imprinted domain in human embryonic and extra-embryonic compartments and its possible role in fetal growth restriction. *Epigenetics*.

[31] Lustig-Yariv O., Schulze E., Komitowski D. (1997). The expression of the imprinted genes H19 and IGF-2 in choriocarcinoma cell lines. Is H19 a tumor suppressor gene?. *Oncogene*.

[32] Arita T., Ichikawa D., Konishi H. (2013). Circulating long non-coding RNAs in plasma of patients with gastric cancer. *Anticancer Research*.

[33] Ji P., Diederichs S., Wang W. (2003). MALAT-1, a novel noncoding RNA, and thymosin *β*4 predict metastasis and survival in early-stage non-small cell lung cancer. *Oncogene*.

[34] Wang Y., Xue D., Li Y. (2016). The long noncoding RNA MALAT-1 is A novel biomarker in various cancers: a meta-analysis based on the GEO database and literature. *Journal of Cancer*.

[35] Spizzo R., Almeida M. I., Colombatti A., Calin G. A. (2012). Long non-coding RNAs and cancer: a new frontier of translational research?. *Oncogene*.

[36] Yu B., Shan G. (2016). Functions of long noncoding RNAs in the nucleus. *Nucleus*.

[37] Ponting C. P., Oliver P. L., Reik W. (2009). Evolution and functions of long noncoding RNAs. *Cell*.

[38] Ding W., Ren J., Ren H., Wang D. (2017). Long noncoding RNA HOTAIR modulates MiR-206-mediated Bcl-w signaling to facilitate cell proliferation in breast cancer. *Scientific Reports*.

[39] Zhou X., Yin C., Dang Y., Ye F., Zhang G. (2015). Identification of the long non-coding RNA H19 in plasma as a novel biomarker for diagnosis of gastric cancer. *Scientific Reports*.

[40] Matouk I. J., Raveh E., Abu-lail R. (2014). Oncofetal H19 RNA promotes tumor metastasis. *Biochimica et Biophysica Acta (BBA)—Molecular Cell Research*.

[41] Qi P., Zhou X.-Y., Du X. (2016). Circulating long non-coding RNAs in cancer: current status and future perspectives. *Molecular Cancer*.

[42] Fanale D., Incorvaia L., Castiglia M. (2017). Liquid biopsy in gastrointestinal stromal tumor. *Liquid Biopsy in Cancer Patients*.

[43] Fayda M., Isin M., Tambas M. (2015). Do circulating long non-coding RNAs (lncRNAs) (LincRNA-p21, GAS 5, HOTAIR) predict the treatment response in patients with head and neck cancer treated with chemoradiotherapy?. *Tumor Biology*.

