# Supplementary material for: Are Long Noncoding RNAs New Potential Biomarkers in Gastrointestinal Stromal Tumors (GISTs)? The Role of H19 and MALAT1
Source: J Oncol. 2019 Nov 15;2019:5458717. doi: 10.1155/2019/5458717 (PMC6885275; doi:10.1155/2019/5458717)
Supplement: Supplementary Materials — Supplementary Table 1: clinical features of the GIST samples. Majority of the patients were males (26; 65%). The median age of the patients was 59 years. Majority of the patients had a mitotic rate <5/50 HPF, tumor size <5 cm, and gastric and intestinal site. [file 5458717.f1.docx]

**Supplementary Material**

**Table 1. Clinical and pathological features of the GIST patients included in this study.**

Majority of the patients consisted of males (26; 65%). Median age of the patients was 59 years. Majority of the patients had a mitotic rate <5/50 HPF, Tumor size <5 cm, gastric and intestinal site.

| **Sex** | Male | 26 |
| --- | --- | --- |
|  | Female | 14 |
| **Median age (years)** |  | 59 |
| **Mitotic rate** | <5/50 HPF | 10 |
|  | ≥5/50 HPF | 12 |
| **Tumor size** | <5 cm | 14 |
|  | ≥5 cm | 9 |
| **Tumor site** | Stomach/Small intestine | 24 |
|  | Colon/Rectum | 2 |
| **Risk classification (Miettinen's criteria)** | Low risk/very low risk | 8 |
|  | Intermediate/high risk | 8 |
